# *Helicobacter pylori* and gastrointestinal and neurological diseases: Study protocol of an umbrella review of systematic reviews and meta-analyses

**DOI:** 10.1097/MD.0000000000018460

**Published:** 2019-12-27

**Authors:** Baoning Wang, Jing Zhang, Sihan Chen, Mingjiang Bie

**Affiliations:** a West China School of Basic Medical Sciences and Forensic Medicine.; b West China School of Public Health and West China Fourth Hospital, Sichuan University, Chengdu, Sichuan; c Jane Lab, Big Data Research Center, University of Electronic Science and Technology of China, Chengdu; d Editorial Board of Journal of Sichuan University (Medical Science Edition), Chengdu, People's Republic of China.

**Keywords:** gastrointestinal diseases, *Helicobacter pylori*, neurological diseases, umbrella review

## Abstract

**Background:**

Systematic reviews showed that *Helicobacter pylori* (HP) infection is a major risk for developing gastric cancer and gastric ulcer and that it might be the cause of inflammatory bowel diseases, functional gastrointestinal disorders, and neurological diseases like Alzheimer disease. However, the robustness of the evidence was not tested. We will perform an umbrella review to systematically evaluate current evidence on the correlation between HP infection and gastrointestinal and neurological diseases.

**Methods:**

We will search OVID MEDLINE, EMBASE, and the Cochrane library for systematic reviews that evaluate the correlation of HP with gastrointestinal and neurological diseases, from inception to 1 July, 2019. Two reviewers will independently screen titles and abstracts of retrieved articles for eligible studies, and they will extract information for data analysis. We will assess heterogeneity between studies using I^2^ statistics and evaluate small-study effect in each systematic review through Egger test. Excess significance bias will be evaluated by compared the expected number of clinical studies with positive findings with the observed number. Quality of each systematic review will be assessed by using AMSTAR2 checklist.

**Ethics and dissemination:**

This umbrella review is anticipated to be finished in December 2019, and the results will be published in a peer-reviewed journal and disseminated through conference presentation or poster. Because all of the data used in this systematic review and meta-analysis has been published, this review does not require ethical approval.

**Registration:** PROSPERO CRD42019137226

## 1 Introduction

*Helicobacter Pylori* (HP), a gram-negative bacterial pathogen, has infected about 50% global population. Global morbidity of HP infection shows significant variability; developing countries tends to have higher infection rate.^[1]^ HP is spread through several ways including person-to-person, oral-oral, faecal-oral, waterborne or iatrogenic routes.^[2]^ HP usually colonize on the human gastric mucosa in early childhood and exist for lifetime. The asymptomatic infection caused by HP in most cases leads to it being overlooked easily

Gastric adenocarcinoma results from HP infection can divided into several phases: gastritis, atrophic gastritis, intestinal metaplasia, atypical hyperplasia and cancer.^[3]^ HP has been classified as a high level cancerogen and has been considered as the main cause of peptic ulcer and stomach cancer. Around 20% of HP-infected patients are infected with gastric, duodenal ulcers and gastric tumors; around 10% chronic active gastritis caused by HP infection progress to severe atrophic gastritis which leads about 5% patients to intestinal gastric cancer.^[4]^ HP infection eradiation has been proved to be the gold standard in peptic ulcer disease treatment and potential to prevent gastric cancer.^[5]^ Gastric adenocarcinoma is the third most cause of cancer-related death, which caused 754,000 people's death in 2015 (World Health Organization, 2016). HP may alter the mucosal barrier directly through modifying the gastric mucins expression.[^6^,^7^,^8^,^9^,^10^,^11^] In recent years, the morbidity of HP infection had decreased due to urbanization and antibiotic usage in childhood. However, the morbidities of stomach cancer and atrophic gastritis changed little and even increased respectively.^[12]^ Increased antibiotic resistance makes the HP infection treatment still a great challenge. Without effective precautionary measures the high morbidity of stomach cancer would keep stable even increase in 2030.^[13]^

The infection of HP is able to result in some chronic diseases such as nervous system diseases (Alzheimer's disease and vascular dementia), Cerebrovascular and cardiovascular diseases, blood disease, skin disease, diabetes and Metabolic syndrome. The infection of HP had been reported to have greatly positive correlation with these diseases.^[14]^ Nervous system diseases became main problems in recent researches among such diseases. The correlation with dementia showed significantly different in related studies, while an initial meta-analysis indicated the HP infection had positive correlation with dementia.^[15]^ The elimination of HP was related to lowering the risk of stomach cancer.[^16^,^17^] Recent meta-analysis founded that even in the high-risk population, the HP eradication was beneficial to patients with atrophic gastritis and intestinal metaplasia.^[18]^

Lots of systematic reviews have studied on the relationship between HP infection and risks of gastric ulcer, inflammatory bowel disease, cholecystitis, stomach cancer and dementia. However, there has been no attempt to review the relationship between infection of HP and all these diseases thoroughly. This research will make a comprehensive review of these systematic reviews and assessed the evidence of correlation with HP infection and different diseases. The possibilities of analyzing the strength of evidence which associated with correlation between HP infection and HP-related diseases morbidity as well as potential deviation degree were provided by the umbrella review. Specifically, present study focused on morbidity of HP-related diseases and aimed to quantify the increased risk of these diseases with HP infection.

## 2 Methods and analysis

This umbrella review protocol was designed according to Preferred Reporting Items for Systematic Reviews and Meta-analyses (PRISMA) and umbrella guideline^[19]^ and it will be reported according to PRISMA.

## 3 Eligibility criteria

We will include participants with any of the following conditions: gastrointestinal tumor (gastric cancer, hepatic tumor, colorectal cancer, and gallbladder cancer), gallbladder diseases, inflammatory bowel diseases (Crohn diseases and ulcerative colitis), functional gastrointestinal disorders (dyspepsia, gastroesophageal reflux, and irritable bowel syndrome), and neurological diseases (Alzheimer disease, dementia, Parkinson diseases, migraine, and stroke); and we will include participants taking tests of HP infection or taking the treatment of eradication of HP infection.

We will screen for systematic reviews that evaluate the correlation of HP infection with gastrointestinal diseases and neurological diseases. Systematic reviews with or without meta-analyses will be included. When multiple systematic reviews focusing on the same condition are found, we will select the most updated one or the one with the largest number of original studies.

## 4 Information sources

We will search MEDLINE, EMBASE, Cochrane Library, and Web of Science (ISI) (from inception to 1 July, 2019) for systematic reviews that evaluate the correlation between HP infection and gastrointestinal and neurological diseases. We will also search PROSERPO (https://www.crd.york.ac.uk/) for reviews on the topic. The search will be performed without language restriction. The complete search strategy is summarized in Table 1.

**Table 1:**
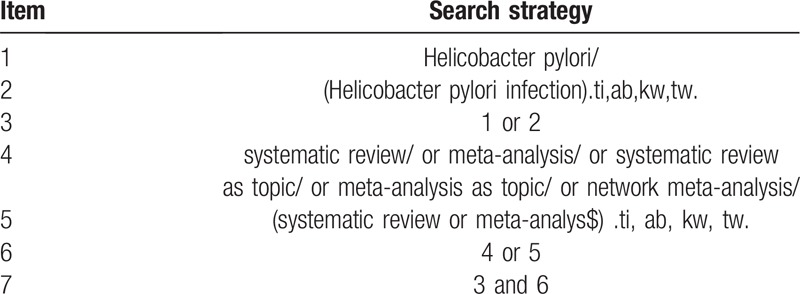
Search strategy (OVID MEDLINE).

## 5 Study selection and data extraction

Study selection and data extraction will be carried out in 3 steps. In step 1, two independent reviewers will screen titles and abstracts identified from the literature search. In step 2, when they could not determine whether an article should be included by screening titles and abstracts, they will further read the full-text copy for additional information. Inter-rater agreement for study inclusion will be calculated using the percent agreement, whereby the study screening process will continue only if > 90% of agreement is observed. If there is any discrepancy (that is, < 90% agreement), it will be resolved through a team discussion. In step 3, two reviewers will independently extract data from included studies. In this phase, the reviewers will independently extract data, including characteristics of systematic reviews (study design, year of study conduct, number of original studies, with or without meta-analyses), patient characteristics (number of participants, mean age, type of diseases, disease subtype, the test for HP, mean duration of disease, accompanied conditions, place of residences (hospital or community settings), quantitative measurements (number of disease events, number of participants with HP infection, number of participants with eradication of HP infection, number of studies with positive findings).

## 6 Methodological quality evaluation

Two reviewers will independently appraise the methodological quality of the included studies using the Measurement Tool to Assess systematic Reviews second version (AMSTAR2).^[20]^ AMSTAR2 is a revised version of AMSTAR, a popular instrument for critically appraising systematic reviews of randomized controlled trials (RCT). The AMSTAR2 has a total of 16 items to assess 7 critical domains of systematic reviews. The 7 critical domains include: protocol registered before commencement of the review (item 2); adequacy of the literature search (item 4); justification for excluding individual studies (item 7); risk of bias from individual studies being included in the review (item 9); appropriateness of meta-analytical methods (item 11); consideration of risk of bias when interpreting the results of the review (item 13); assessment of presence and likely impact of publication bias (item 15). We will classify the quality of included systematic reviews into one of the four levels: high, moderate, low, or critically low. High confidence refers to systematic reviews without non-critical weakness and systematic reviews that provide accurate and comprehensive summaries of available studies that address the question of interest. Moderate confidence refers to systematic reviews with more than one non-critical weakness. Low confidence refers to systematic reviews with at least one critical flaw. Critically low confidence refers to systematic reviews with more than one critical flaw.

## 7 Outcome assessments

The primary outcome will be the risk ratio of gastrointestinal or neurological diseases after HP infection. Secondary outcomes include the risk ratio of gastrointestinal tumor (gastric cancer, hepatic tumor, colorectal cancer, and gallbladder cancer), gallbladder diseases, inflammatory bowel diseases (Crohn diseases and ulcerative colitis), functional gastrointestinal disorders (dyspepsia, gastroesophageal reflux, and irritable bowel syndrome), and neurological diseases (Alzheimer disease, dementia, Parkinson diseases, migraine, and stroke) after HP infection,

## 8 Synthesis of included studies

This protocol will summarize the main findings of the eligible systematic reviews. For systematic reviews with meta-analysis, we will use random-effects model (meta package in R 3.5.0) calculate the summary ES and 95%CI. We will estimate the 95% prediction intervals (PIs) and assess whether they excluded null value. We use I^2^ statistics to assess the between-study heterogeneity in each meta-analysis. We classified the heterogeneity as 3 degrees: small (I^2^ < 25%), moderate (25% ∼ < I^2^ < 50%), and large (I^2^ > 50%). And use the Egger test to evaluate publication bias and small-study effect. To evaluate the excessive significant bias, we will run a test to assess whether the observed number of studies (O) with significant results (positive studies with *P* < .05) is larger than their expected number (E). E is calculated by the sum of the actual power of each original study, the true effect size of HP infection will be estimated through the parameters of the original study with the largest sample size in a meta-analysis. We will also calculate the O/E ratio to evaluate the extent of excess significance bias, and assess the statistical significance of the bias through Chi-squared test; when a *P* < .05 is reached, we will consider the existence of significant bias.

The evidence of the correlation between HP infection and gastrointestinal and neurological diseases will be categorized into strongest-validity, highly suggestive, suggestive, or weak evidence according to the criteria.^[21]^ The evidence with strong-validity will fulfill:

(1) *P* value < .05 in fixed-effects model or had *P* value < .001 in random-effects model;
(2) at least 1000 participants;
(3) low or moderate between-study heterogeneity (I^2^ < 50%);
(4) 95%PI that excludes the null value;
(5) no evidence of small-study effects and excess significance bias.

The highly suggestive evidence meet criteria (1) to (4); the suggestive evidence meet (1) and (2); the weak evidence will meet only (1).

## 9 Discussion

As far as we know, none research has been reported about comprehensive estimate of the relationships between HP infection and gastrointestinal and neurological diseases. This review aims to identify the primary outcome of risks in these patients and will be published in peer-review journal and conference proceedings. Moreover, GRADE method has been adapted in our research to evaluate the strength of evidence.

Our research is able to provide a new commentary on the current systematic review evidence for the association between HP infection and the risks of gastrointestinal and neurological diseases. It provides an accessible, comprehensive results with which to inform clinicians and to develop the guidelines for the management of the individuals with HP infection. Additionally, it is expected that this umbrella review will encourage further research to clarify the relationships for which systematic review evidence of high quality is currently insufficient or lacking.

## Author contributions

**Conceptualization:** Baoning Wang.

**Data curation:** Baoning Wang, Jing Zhang.

**Formal analysis:** Baoning Wang, Jing Zhang.

**Project administration:** Sihan Chen.

**Supervision:** Mingjiang Bie.

**Writing – original draft:** Baoning Wang.
